# Combining pneumatic ballistic lithotripsy with an ultrasonic lithotripsy system in pancreatic duct stone surgery: A case report with video

**DOI:** 10.1097/MD.0000000000044139

**Published:** 2025-08-29

**Authors:** Zhen Wang, Jun Chen, Dao-Jun Gong

**Affiliations:** a Department of Hepatobiliary Pancreatic Surgery, Jinhua Hospital of Zhejiang University, Jinhua, Zhejiang Province, China.

**Keywords:** chronic pancreatitis, EMS lithotripsy, pancreatic duct stones, Partington procedure, surgery

## Abstract

**Rationale:**

Chronic pancreatitis can lead to the development of pancreatic stones. These stones may cause obstruction of the pancreatic duct, resulting in elevated intraductal pressure and abdominal pain. Surgery is an effective treatment for pancreatic stones. However, the removal of large pancreatic duct stones often presents significant technical challenges.

**Patient concerns:**

An 18-year-old female patient was admitted due to upper abdominal pain and recurrent vomiting over a 3-month period.

**Diagnoses:**

Abdominal computed tomography showed multiple stones throughout the pancreatic duct and an exceptionally large stone (20 mm diameter) incarcerated in the main pancreatic duct.

**Interventions:**

The pancreatic duct was incarcerated within the main pancreatic duct and could not be removed. EMS lithotripsy was used to fragment and aspirate the stones.

**Outcomes:**

Intraoperative exploration confirmed the absence of visible stone residues. The patient remained pain-free during the one-year postoperative follow-up.

**Lessons:**

EMS lithotripsy may serve as an effective alternative for patients with multiple pancreatic duct stones who are not candidates for endoscopic treatment and for whom the removal of impacted stones during surgery proves challenging.

## 1. Introduction

Pancreatic duct (PD) stone formation is a common pathological change that occurs during the course of chronic pancreatitis, with an incidence of >90%.^[1]^ An endoscopic approach via endoscopic retrograde cholangiopancreatography may be preferred for patients with uncomplicated obstructions located in the head, neck, or body of the pancreas. However, surgical intervention should be considered as a first-line option when endoscopic treatment is likely to be ineffective, such as in cases involving disease in the tail of the pancreas or significant calcification and stone burden that are not amenable to endoscopic management.^[2]^ Partington’s procedure is still the first choice for patients with dilation of the main PD, but no inflammatory pancreatic head mass.^[3,4]^ When selecting surgical treatment, it may occur that the main PD stone is too large to be removed during the procedure. Herein, we report the successful removal of an incarcerated giant stone using EMS lithotripsy.

## 2. Case report

**Figure 1.**
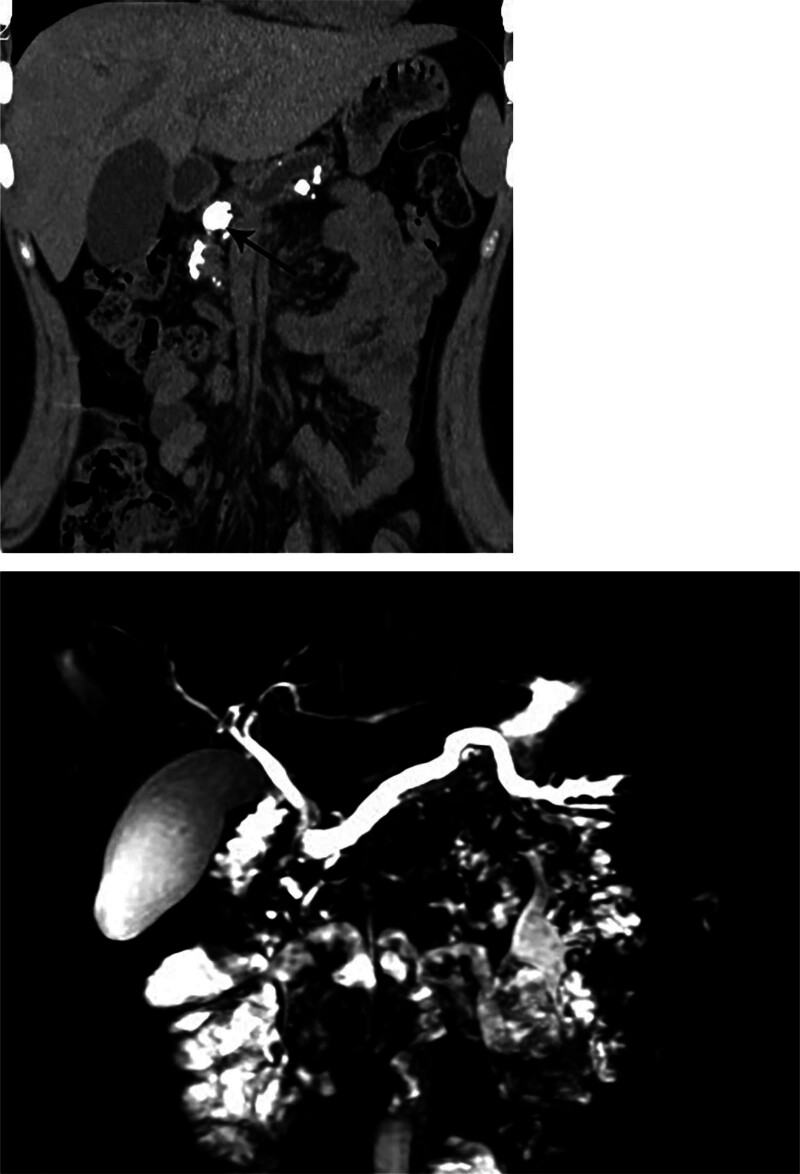
A computed tomography scan of the patient’s abdomen shows a dilated pancreas with an abnormally large stone embedded in the main pancreatic duct (indicated by an arrow).

An 18-year-old female patient was admitted because of upper abdominal pain and recurrent vomiting over a 3-month period. After consuming solid food or semiliquid food, the patient experienced pronounced nausea and vomiting, along with a weight loss of 6 kg. There had no relevant family history or history of alcohol consumption or smoking. On physical examination, the vital signs were as follows: body temperature, 36.6℃; blood pressure, 117/69 mm Hg; heart rate, 86 beats/min; and respiratory rate, 20 breaths/min. There were no positive signs, except for tenderness in the epigastric region. Laboratory tests including hemoglobin, amylase, C reactive protein, gamma-glutamyl transpeptidase and CA 19 to 9 were normal. Computed tomography and magnetic resonance imaging revealed pancreatic atrophy, dilation of the PD, and multiple nodular high-density shadows within the pancreas (Fig. 1).

**Figure 2.**
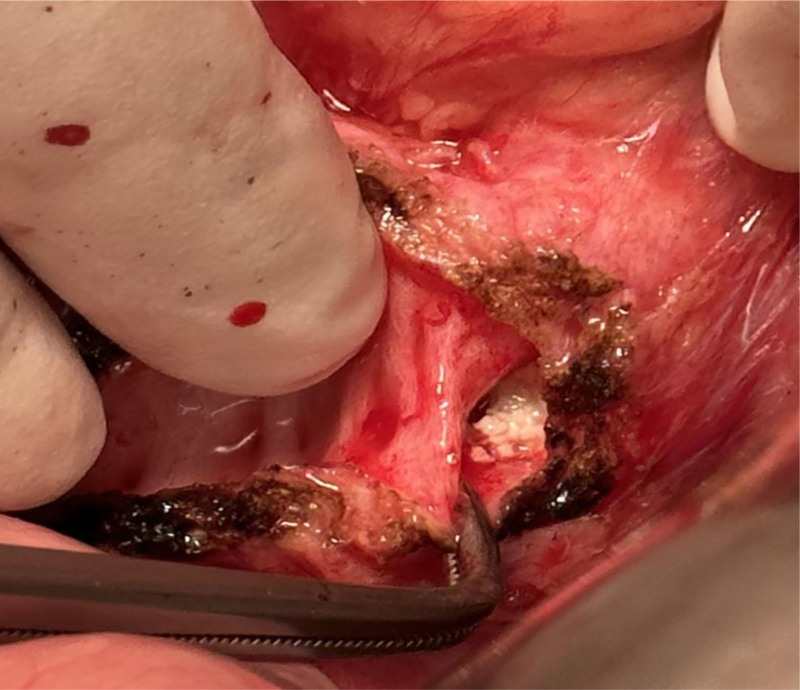
Intraoperatively encountered major giant pancreatic duct incarcerated stone.

**Figure 3.**
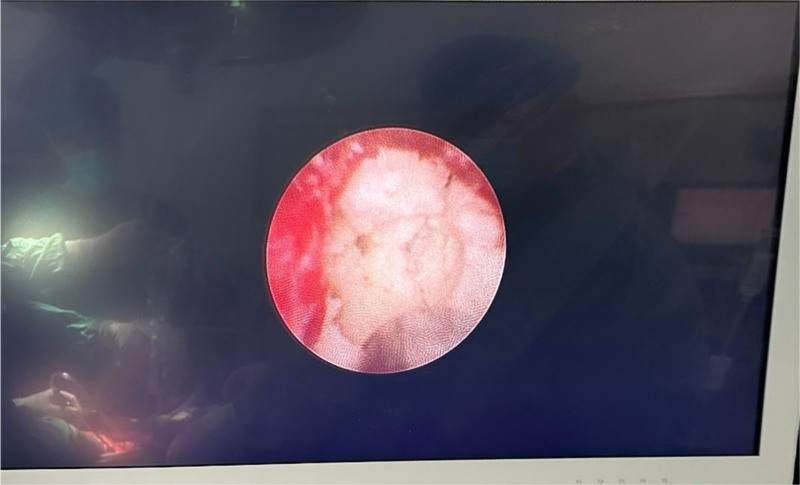
EMS lithotripsy screen display.

**Figure 4.**
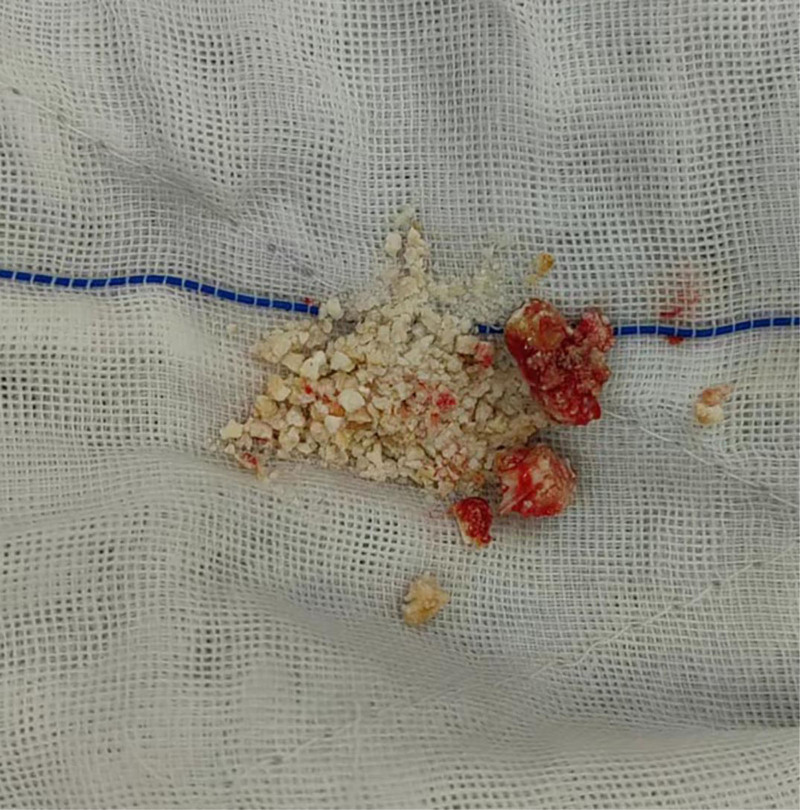
Intraoperative stone removal samples were collected.

Surgical procedure: A midline incision (approximately 15 cm) was made on the upper abdomen. An incision was made on the surface of the pancreas, and the main PD was identified. Using an electrocautery device, we extended the duct from the pancreatic head to tail. Multiple stones were observed in the main PD of the head. The stones had a hard texture and the largest stone was 20 mm in diameter. It was incarcerated within the main PD (Fig. 2) and could not be removed using the vascular forceps. Assisted by urologists, EMS lithotripsy (Electro Medical Systems, Rte. De Champ-Colin 2, 1260 Nyon, Switzerland, IMPACT = 45%, FREQUENCY = 8 Hz) was used to fragment and aspirate the stones (Fig. 3). Intraoperative exploration with the EMS lithotripsy probe confirmed the absence of visible stone residues in both the main PD and the PD branches. The proximal jejunum was lifted, and a straight-cut closure device was used to secure it 25 cm from the ligament of Treitz. The distal jejunum was lifted and sutured side-to-side with the main PD to complete pancreaticojejunostomy. An end-to-side anastomosis was established between the jejunum and proximal jejunum, approximately 50 cm away from the pancreaticojejunostomy, with drainage tubes situated both above and below the anastomosis. The stones removed during surgery are shown in Figure 4. The procedure was completed within 200 minutes, with an estimated intraoperative blood loss of approximately 50 mL. No complications occurred during or after the surgery.

**Figure 5.**
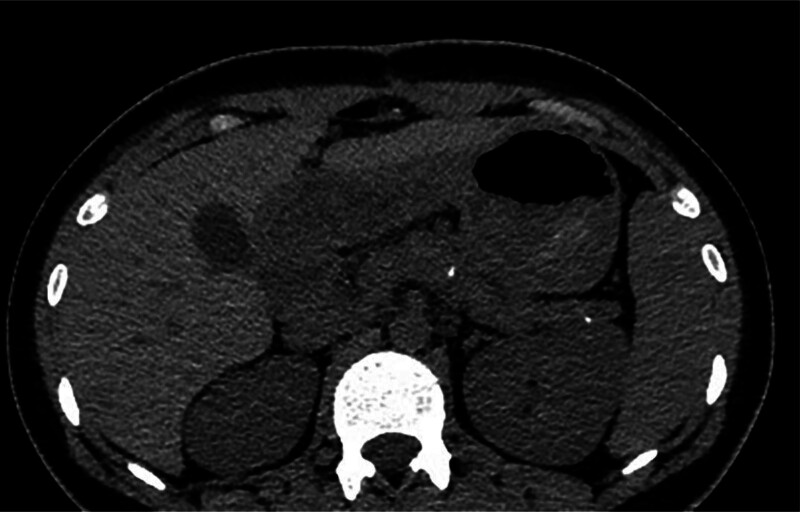
CT reexamination 1 year after surgery. CT = computed tomography.

The patient reported no abdominal pain on the fifth day post-operation. The upper anastomotic drainage tube was removed on the seventh day, and the patient was discharged on the 10th postoperative day. The lower anastomotic drainage tube was removed on the 14th day. The patient remained pain-free during the 1-year postoperative follow-up, and computed tomography revealed no newly formed PD stones (Fig. 5).

## 3. Discussion

During chronic pancreatitis, the development of PD stones can aggravate obstruction within the PD, resulting in elevated intrapancreatic pressure and subsequent ischemic conditions. Consequently, removal of PD stones is crucial to effectively alleviate the symptoms associated with chronic pancreatitis. For patients presenting with irregularly configured main PD stones and multiple branching PD stones, extracorporeal shock wave lithotripsy and endoscopic stone extraction frequently necessitate repeated stone removals, PD dilations, and even multiple insertions of stent tubes. These procedures increase risks and extend the patient’s recovery period. In contrast, single-stage surgical interventions have distinct advantages in managing such cases.^[5–7]^ At the same time, extracorporeal shock wave lithotripsy requires precise localization of the stones, and is not suitable for pancreatitis patients with widely distributed stones, or for those with isolated stones in the pancreatic tail, because it heightens the risk of spleen injury. Two other randomized controlled trials comparing surgical intervention with endoscopic treatment in patients with long-standing chronic pancreatitis and opioid dependence demonstrated that surgery was more effective than endoscopic treatment in alleviating pain.^[6,8]^Another recent multicenter study demonstrated that patients who receive short-term opioid therapy prior to surgery experience better analgesic outcomes following surgical intervention compared to endoscopic treatment. In patients with chronic pancreatitis, early surgical intervention provides superior pain relief compared to delayed surgery.^[9]^ Based on the aforementioned research findings, we selected the Partington procedure, which is associated with minimal surgical complications and low mortality rates. This approach not only alleviates pain but also preserves the endocrine and exocrine functions of the pancreas more effectively. In the preoperative assessment, we predicted that the stones would be difficult to remove; therefore, we sought suitable equipment for this purpose. According to Sahoo et al, pancreatic tail stones can potentially be removed using a cystoscope in conjunction with an endoscopic basket.^[10]^ Eventually, we selected EMS lithotripsy. This is because relevant research has demonstrated that compared with traditional lithotriptors, EMS lithotripsy features simpler and more efficient operation procedures, a more potent ability to fragment calculi thoroughly, and can effectively aspirate the fragmented calculi.^[11–16]^

## 4. Conclusion

Through this case, our team has drawn the conclusion that EMS lithotripsy can aid pancreatic surgeons in more effectively addressing PD stones in patients with complex large main duct stones, branch duct stones, and tail-of-pancreas stones. The preliminary results indicate both safety and efficacy; however, additional large-scale clinical trials are required to comprehensively assess its long-term efficacy and safety profile.

Supplemental Digital Content “Video” is available for this article.

## Author contributions

**Writing – original draft:** Zhen Wang.

**Writing – review & editing:** Jun Chen, Dao-Jun Gong.
